# The Training of Officers

**Published:** 1908-02-08

**Authors:** 


### The Royal Army Medical Corps Section.

### THE TRAINING OF OFFICERS.

The Royal Army Medical Corps, according to the official Manual, is organised for the performance of duties in connection with the hospital and ambulance service of the army. These duties are very varied, and the corps is responsible, again to quote the Manual, both in peace and war, at home and abroad, not only for the nursing of the sick, but also for equipment; requisition of fuel, light, provisions, and all requisite supplies and repairs; the cooking and expenditure of diets; the custody of patients' kits; the cleanliness of the hospital and its surroundings, the exchange of bedding, etc., and the preparation of the necessary accounts, abstracts, and vouchers of expenditure. In addition, there is the duty in the field connected with sick and wounded, field hospitals, convoys, hospital trains, stationary and general hospitals.

To enable the men of the R.A.M.C. to undertake even the most elementary of their duties either in hospitals or in the field, it follows as a matter of necessity that they must undergo a course of technical training. If this be so with regard to the men, it applies with increased force to the officers who have charge of these men, who "must take every opportunity" according to the Regulations, "of instructing them in their various duties," and whose responsibilities are, therefore, proportionally greater. For the officers of the R.A.M.C. an elaborate course of training is prescribed so that they may acquire a practical knowledge of the management of hospitals and the interior economy and training of the corps. If, therefore, the Territorial Army Medical Service is to be an exact counterpart of the R.A.M.C. and its officers are to take their proper places, and take them with any semblance of efficiency, it follows that they also will require opportunities for instruction in the duties which will be required of them. Each officer should, in fact, be, so far as is feasible, an exact counterpart of his neighbour of the R.A.M.C. He is, in fact, an understudy.

The manifest difficulty to be faced lies in the fact that the territorial officer has other duties in time of peace, duties upon which his livelihood probably depends, and which must, therefore, be paramount. The time at his disposal is accordingly limited, and if the service of the country demands his proficiency it is but reasonable to expect that the requisite instruction be given at times suitable and convenient for the pupil. We have referred in a previous article to the lack of facilities for the efficient training of the men, but the officers have in the past been in worse plight. Hitherto an officer on joining the Volunteer Medical Service was compelled within a period of two years to pass an examination in certain subjects. But he was left almost entirely to his own resources as to the method of obtaining the necessary knowledge to enable him to pass successfully. As a result many officers considered their medical knowledge sufficient for the purpose, and attended before examination boards when 60 per cent. failed!

There is a prescribed course for officers who can attend at Aldershot, but as it extends over a period of six weeks the absurdity of expecting a medical man to leave his practice for such a prolonged training ought to be apparent. Recently a step has been taken in the proper direction by instituting at provincial military hospitals a course of instruction of a fortnight's duration, particularly for selected officers in view of promotion to majority. The course is an excellent one, comprehensive in character, and of practical value, and were officers encouraged to attend a similar course on enrolment much benefit would undoubtedly accrue. The question of pay is of secondary importance at this stage. The facilities for the training, in view of the compulsory proficiency examination, ought to be afforded to every officer if more than a mere paper proficiency is desired.

It may be of interest to detail this course of instruction as recently carried out at one of the military station hospitals in the provinces. For the metropolis there has never been any considerable difficulty as a private school of instruction exists there; but the provincial officer has lacked the opportunities and the encouragement now given. In the newly constituted course the number of officers in attendance is limited in order to ensure that each individual receives instruction in the ordinary routine of the work. The lectures and demonstrations occupied two hours each day, from 10 A.M. till noon. The first lecture dealt with the history of the sick soldier, mode of admission to and discharge from hospital, the documents of the soldier in hospital, and the forms and books connected with his admission, stay, and discharge. The first demonstration was of forms, books, pack-store, and hospital clothing-store.

Lecture II. dealt with hospital administration in peace; diets, medicines, linen, washing, ward system and the relation of officers, nurses, and orderlies to each other, and to the officer in charge. The demonstration was of ward work, and of the daily, monthly, and annual returns.

Lecture III. dealt with discipline in connection with the corps and with the patients. The demonstration consisted of attendance at orderly room, and explanation of the various forms and books there, and the work carried on.

Lecture IV.: Interior economy of the army. Demonstration: Kit inspection, care of barrack-rooms, messing, clothing, etc.

Lecture V.: Barrack life and sanitation, with reference to the King's regulations; medical regulations as to messing, etc. The officers attended at barrack-room inspection by the medical officer in charge of the troops.

Lecture VI.: Recruits and recruiting regulations. A full period of two hours was spent on the following day at the local recruiting office where the medical examination of recruits was witnessed in detail.

Lecture VII.: Training of the soldier, effects of drills and exercises, gymnasia, etc. The officers then attended the gymnasium of the station and witnessed the physical drill under the instructors.

Lecture VIII.: Supplies for men in barracks and hospital; conditions of contract; examination of provisions. The officers attended the ration-stands in barracks, hospital, stores, and kitchens, and were instructed in the methods adopted. A demonstration of the regulation filter-cart was also given. Two lectures were then given on army medical organisation in war, the constitution of divisions, brigades, and other units with the medical personnel for each part of the field army; also the medical organisation at the base and on the lines of communication.

The lectures were devoted to the duties and rôle of field ambulances, stationary and general hospitals. Two lectures were given on sanitation in war in relation to camps, bivouacs, and the duties of medical officers in connection therewith, and a final lecture dealt with the Geneva Convention and the relation of voluntary aid to an army in war.

The officers attending had thus an opportunity of obtaining both theoretical and practical instruction in the whole duties of a medical officer. They saw the regular routine of ward work, inspection of barracks, passing of recruits, and treatment of sick, and they saw this without any serious disorganisation of their own work or of the work of the station. Such courses deserve every encouragement, as they tend to bring the officer into touch with the duties which will be required of him, should the necessity for the mobilisation of the Territorial Army arise. The work will be in great measure necessary even without actual war; the mobilisation of a civilian army will necessitate very special medical supervision. There will be no place for amateurs; there will be work to be done, and only proficient officers can do it with any chance of success.

We trust that the proposed "schools of instruction" to be created throughout the country will receive every encouragement at the hands of the authorities; that they will be so equipped that a satisfactory course can be obtained; and that the officers who will fill the rôle of instructors will be capable of doing so to the benefit of the pupils. We say this advisedly. Competent instruction can only be secured through competent instructors, and in the medical service, above all, the work to be undertaken should be undertaken in earnest.

